# Epidemiological Trends of Antibiotic Resistant Gonorrhoea in the United Kingdom

**DOI:** 10.3390/antibiotics7030060

**Published:** 2018-07-13

**Authors:** Lilith K. Whittles, Peter J. White, John Paul, Xavier Didelot

**Affiliations:** 1 Department of Infectious Disease Epidemiology, School of Public Health, Imperial College London, London W2 1PG, UK; l.whittles14@imperial.ac.uk (L.K.W.); p.white@imperial.ac.uk (P.J.W.); 2 MRC Centre for Outbreak Analysis and Modelling, School of Public Health, Imperial College London, London W2 1PG, UK; 3 NIHR Health Protection Research Unit in Modelling Methodology, School of Public Health, Imperial College London, London W2 1PG, UK; 4 Modelling and Economics Unit, National Infection Service, Public Health England, London NW9 5EQ, UK; 5 Department of Microbiology, Public Health England Collaborative Centre, Royal Sussex County Hospital, Brighton BN2 5BE, UK; John.Paul@phe.gov.uk; 6 Department of Global Health and Infection, Brighton and Sussex Medical School, University of Sussex, Falmer BN1 9PH, UK

**Keywords:** gonorrhoea, antibiotic resistance, cephalosporins, azithromycin, sexually-transmitted bacterial infections

## Abstract

Gonorrhoea is one of the most common sexually-transmitted bacterial infections, globally and in the United Kingdom. The levels of antibiotic resistance in gonorrhoea reported in recent years represent a critical public health issue. From penicillins to cefixime, the gonococcus has become resistant to all antibiotics that have been previously used against it, in each case only a matter of years after introduction as a first-line therapy. After each instance of resistance emergence, the treatment recommendations have required revision, to the point that only a few antibiotics can reliably be prescribed to treat infected individuals. Most countries, including the UK, now recommend that gonorrhoea be treated with a dual therapy combining ceftriaxone and azithromycin. While this treatment is still currently effective for the vast majority of cases, there are concerning signs that this will not always remain the case, and there is no readily apparent alternative. Here, we review the use of antibiotics and epidemiological trends of antibiotic resistance in gonorrhoea from surveillance data over the past 15 years in the UK and describe how surveillance could be improved.

## 1. Introduction

The threat of antibiotic-resistant gonorrhoea is of grave concern. The causative agent of gonorrhoea is the bacterium *Neisseria gonorrhoeae*, an obligate human pathogen, whose existence is suggested by texts from as early as Leviticus to the writings of Herodotus in the Fifth Century BCE [1]. *N. gonorrhoeae* has developed resistance to each antibiotic used against it over the past 80 years, starting with sulphonamides and penicillin in the 1930’s and 1940’s [2,3]. Consequently, *N. gonorrhoeae* was listed in 2013 as one of the three most urgent antibiotic-resistant threats in a CDC report [4] and in 2018 as a priority pathogen by the WHO [5]. Ceftriaxone is both the first-line treatment, as well as the last remaining single-drug option, and susceptibility is diminishing rapidly [6]. As a result, the United Kingdom, along with most other European countries and the USA, currently recommend dual treatment of gonorrhoea diagnoses with ceftriaxone and azithromycin [7,8,9]. However, the WHO Gonococcal Antimicrobial Surveillance Programme has reported high levels of azithromycin resistance in several countries [10], and even in countries like the UK, where resistance is relatively low, outbreaks of azithromycin-resistant gonorrhoea have recently occurred [11,12], Consequently, the dual-antibiotic regime may not be effective in the long term. In 2014, the worldwide-first case of dual-therapy failure was reported in the UK [13], followed in February 2018 by a similar case that also failed to respond to the last-resort therapy spectinomycin [14].

The UK has collected surveillance data on sexually-transmitted infections, including gonorrhoea, since the Venereal Diseases Act of 1917, which reveal a long and varied history of the disease [15]. The number of diagnoses peaked at over 50,000 cases per year after World War II, before the epidemic was brought under control by the advent of the antibiotic era. Changing sexual attitudes and behaviour in the 1960’s reversed the downward trend, resulting in a second peak of over 60,000 diagnoses per year in the 1970’s. Fears around HIV and the adoption of safer sexual practices caused a decline in gonorrhoea rates throughout the 1980’s and early 1990’s. Beginning in the late 1990’s, increases in sexual risk behaviour led to an increase in incidence, which was exacerbated by a vicious circle in which overstretched sexual health services resulted in reduced access to care, further promoting transmission of infection [16]. Substantial investment in sexual health services in the early 2000’s reversed the trend. However, in recent years, there has been another resurgence in the epidemic, and once again, health services are struggling to cope. This epidemic is much larger than 15 years ago and is aggravated by the threat of antimicrobial resistance [17]. In 2017, over 44,000 cases of gonorrhoea were diagnosed in England, making it one of the most common sexually-transmitted infections. Incidence has risen an average of 12% per year since 2008, from 29–81 cases per 100,000 people [18]. The current epidemic is still far smaller than those seen in the 20th Century when accounting for population growth during that period, which suggests that it could potentially become much larger without effective control measures.

**Figure 1:**
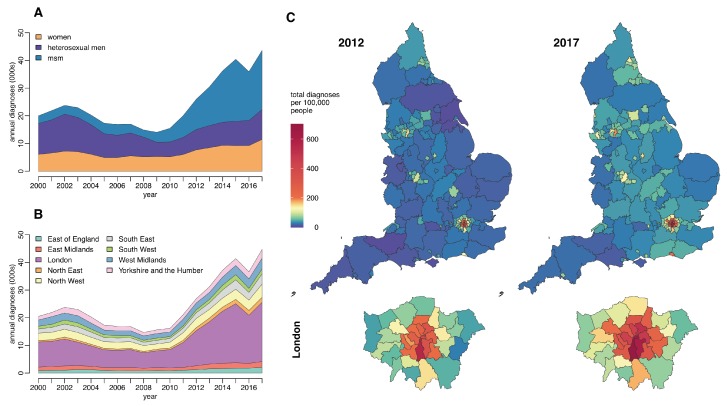
Total annual gonorrhoea diagnoses in England split by (**A**) gender and male sexual behaviour (**B**) and region (**C**) Comparison of the rate of gonorrhoea diagnosis per 100,000 people across regions of England and London boroughs in 2012 and 2017. Data on sexually-transmitted infection diagnoses and rates published by Public Health England (PHE) and accessed using the Fingertips R package [18].

Gonorrhoea tends to cause local outbreaks, spreading quickly when it becomes introduced into clustered sexual networks. Around half of all gonorrhoea infections are diagnosed in Men who have Sex with Men (MSM) (Figure 1A). Young black heterosexuals are disproportionately likely to be affected [19,20], an effect that is not fully explained by recorded sexual risk behaviour [21]. The geographic and socio-cultural distribution of infection in the UK is highly variable and characterised by endemic areas and epidemic clusters (Figure 1B,C) [22]. The incidence in some London boroughs is eight-times greater than the national average (Figure 1C) [19].

**Figure 2:**
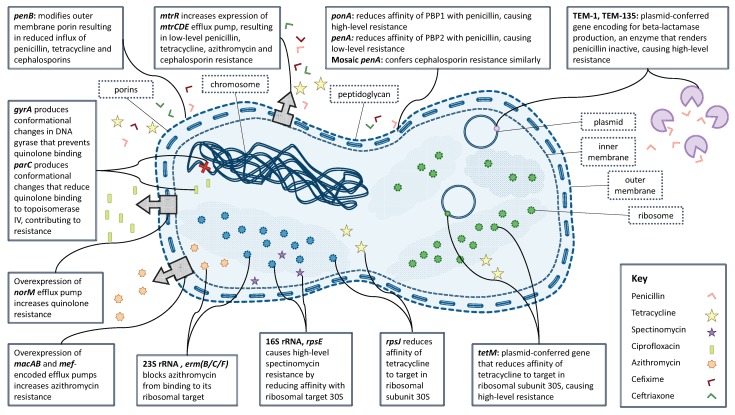
Known mechanisms of resistance in *N. gonorrhoeae* to clinically-relevant antibiotics.

The facility with which *N. gonorrhoeae* is able to alter its genome [23,24,25] has contributed to the emergence and rapid dissemination of antimicrobial resistance. Gonococci are easily able to acquire genetic material from other lineages, and even other species, through both transformation (uptake from the environment) and conjugation (transfer for example of a plasmid between bacterial cells in direct contact) [26,27,28]. Mosaic alleles acquired from other *Neisseria* species have been shown to decrease susceptibility to azithromycin [29], and plasmid-borne resistance to both penicillin and tetracyclines has long been observed [30,31]. *N. gonorrhoeae* has developed, or acquired, resistance mutations via all known physiological mechanisms: the production of antibiotic-destroying enzymes, the modification of antimicrobial targets and decreased influx and increased efflux of antimicrobials (Figure 2) [6]. The evolutionary clock rate of *N. gonorrhoeae* is estimated to be around three substitutions per genome per year, and the relative effect of recombination compared to mutation is around *r*/*m* = 2 [25,32,33]. Neither of these two values is exceptional when compared with other bacterial species [34,35], so the rapid evolution and spread of antibiotic resistance mechanisms is likely explained by other factors of within-host adaptability [36].

## 2. Surveillance of Gonococcal Antibiotic Resistance in the UK

Public Health England (PHE) runs the Gonococcal Resistance to Antimicrobials Surveillance Programme (GRASP), which has produced a report annually since 2000 [37,38,39,40,41,42,43,44,45,46,47,48,49,50,51,52,53]. GRASP monitors trends in resistance and susceptibility of gonorrhoea in England and Wales to antibiotics previously used, currently used or that could one day be used, namely: ceftriaxone, azithromycin, cefixime, ciprofloxacin, tetracycline, penicillin and spectinomycin. These data are used to inform national treatment guidelines and strategies.

All data and figures shown below are based on figures quoted in the GRASP reports or, where exact figures are not available, digitised plot data extracted using Plot Digitizer 2.6.8. While the GRASP definitions of resistance have varied from year to year, we adopt the Minimum Inhibitory Concentration (MIC) breakpoints used in the 2016 GRASP report, which is aligned with EUCAST, unless otherwise stated (Table 1).

**Figure 3:**
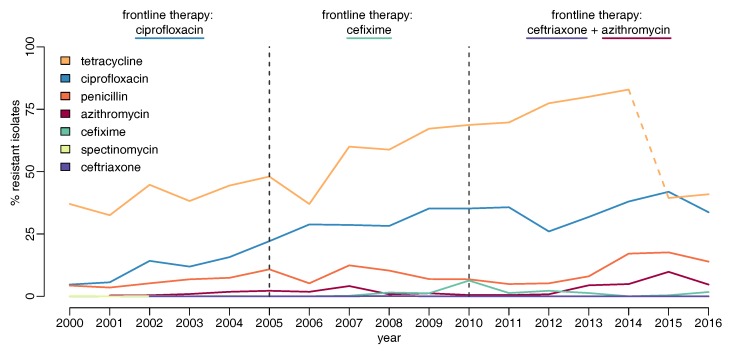
Proportion of gonococcal isolates in Gonococcal Resistance to Antimicrobials Surveillance Programme (GRASP) showing resistance to different antibiotics over time. Dashed vertical lines show dates of treatment guideline change. In 2015, the GRASP agar medium used to determine MIC values changed, so results after this date are not comparable to previous years, particularly for azithromycin and tetracycline. The azithromycin resistance levels for 2013 and 2014 have been adjusted upwards from those published by GRASP, as previously suggested [54], but on the other hand, the tetracycline levels are not directly comparable before and after 2015, as highlighted by the dashed line.

The proportion of GRASP isolates that were susceptible to all clinically-relevant antibiotics fell from 80% to 46% between 2004 and 2015 [54]. Gonococcal resistance to tetracycline, ciprofloxacin and penicillin is high, with resistance levels in 2016 at 41%, 34% and 14%, respectively (Figure 3) [53]. Resistance to cefixime peaked at 6% in 2010, but has fallen to 2% since cefixime was removed from the recommended first-line treatments. Resistance to one of the current first-line treatments, azithromycin, has increased dramatically to 5–10% in 2015–2016; resistance to the other component of dual therapy, ceftriaxone, has been detected only sporadically since 2005. However, it will be very important to monitor resistance trends in these first-line antibiotics going forward, particularly in light of recent reports of dual-therapy treatment failure [13,14,55].

### 2.1. Penicillin

The first use of penicillin to treat gonorrhoea was in 1930 by microbiologist Cecil Paine, who used a crude extract of penicillin-producing fungus to cure a baby with an ophthalmic infection in Sheffield [56]. By 1943, penicillin was being used to treat sulphonamide-resistant urethral gonorrhoea and gradually became the primary treatment [57]. Over the following 30 years, the MIC of penicillin gradually crept upwards due to disseminating chromosomal resistance [6]. The year 1976 marked the beginning of the end of the penicillin-era in gonorrhoea treatment, when a plasmid-mediated β-lactamase-producing strain was identified in Liverpool [30]. High-level chromosomally-mediated resistance emerged in the USA ten years later and quickly spread worldwide [58].

**Figure 4:**
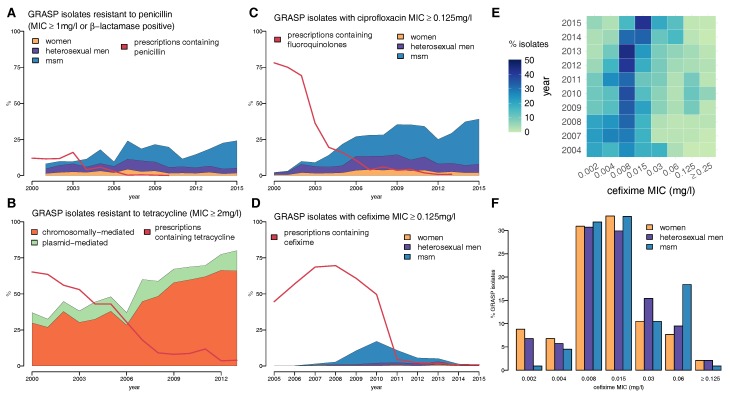
Proportion of gonococcal isolates in GRASP over time showing (**A**) resistance to penicillin, split by gender and male sexual behaviour, compared with the proportion of prescriptions containing penicillin; (**B**) chromosomally- vs. plasmid-mediated tetracycline resistance, compared with the proportion of prescriptions containing a tetracycline; (**C**) resistance to ciprofloxacin by gender and male sexual behaviour, compared with the proportion of prescriptions containing a fluoroquinolone, such as ciprofloxacin or ofloxacin; (**D**) resistance to cefixime by gender and male sexual behaviour; (**E**) cefixime MIC distribution over time and (**F**) cefixime MIC distribution in 2014 split by gender and male sexual behaviour.

When the GRASP programme was initiated in 2000, penicillin was still being prescribed in 12% of cases [37], with 9.5% of isolates being resistant [43]. Resistance has generally been much higher in MSM than in heterosexuals, peaking in 2007 when 40% of MSM had penicillin-resistant gonorrhoea, compared to 16% of heterosexual men and 9% of women (Figure 4A) [52].

### 2.2. Tetracycline

Tetracyclines were discovered in 1945 and first prescribed to treat gonococcal infections in individuals allergic to penicillin [57]. The widespread use of tetracyclines for the treatment of bacterial sexually-transmitted infections, particularly chlamydia, has been implicated in rising resistance [57]. Chromosomally-mediated resistance of gonorrhoea to tetracycline was documented as early as 1957 [59]. Plasmid-mediated tetracycline resistance was first identified in the USA in 1983 and followed two years later by the discovery of a strain in the Netherlands that produced β-lactamase, making it additionally resistant to penicillin [31,60]. By 1988, plasmid-mediated tetracycline-resistant gonorrhoea had arrived in the UK [61].

In 2000, tetracycline resistance was already widespread in UK gonorrhoea at 37% [44] and has since continued to rise, peaking at 83% in 2014 [53]. Although past resistance levels may have been overstated, the sudden drop in detected resistance in 2015 is an artefact of the change in the agar medium used in the GRASP protocol (Figure 3) [52]. The majority of the resistance seen in the UK has been chromosomally-mediated (Figure 4B), and this has generally been more prevalent among MSM. *N. gonorrhoeae* with a mutation in the *mtr* locus, which confers reduced susceptibility to penicillin, tetracycline, azithromycin and cephalosporins, has a cell wall that is resistant to hydrophobic molecules and is thus well adapted to the rectal environment [62], which partly explains why it is more often found in MSM [44].

### 2.3. Ciprofloxacin

Fluoroquinolones were introduced in the early 1980s and were widely welcomed as a replacement for penicillin in the treatment of gonorrhoea, due to their minimal side effects, single-dose formulation and efficacy at all infection sites, including the pharynx [57]. The first report of treatment failure at a 250-mg dosage came from London in 1990, after which the recommended dosage was increased to 500 mg [63]. However, reports of treatment failure at this increased dosage came from Japan less than five years later [6,64].

When GRASP began in 2000, ciprofloxacin and ofloxacin were the recommended first-line treatments for gonorrhoea and were prescribed in 80.2% of cases [41]. Ciprofloxacin-resistant gonorrhoea, at the time defined as having MIC ≥ 1 mg/L, was detected in the first annual report with a prevalence of 2% [44]. If the current resistance breakpoint of MIC ≥ 0.125 mg/L had been used, then prevalence of resistance would have been just below the 5% threshold set by the WHO [53,65].

Ciprofloxacin resistance quickly spread in the UK, and by 2002, the WHO threshold had been breached across all gender and sexuality categories, even at the more stringent MIC breakpoint: 7% of women, 12% of heterosexual men, and 9% of MSM tested positive for resistance (Figure 4C) [66]. Ciprofloxacin was removed from the treatment guidelines in 2005, by which time 22% of GRASP isolates were resistant [53]. Despite the subsequent decline in fluoroquinolone use, ciprofloxacin resistance remains high in the UK with a level of 34% as of 2016 [53].

### 2.4. Cefixime

Cephalosporins were discovered in 1948 and have been chemically modified through three generations to create antimicrobial agents such as cefixime and ceftriaxone, both of which are effective against *N. gonorrhoeae* [57]. The rise in prescribing of cephalosporins has coincided with the decline in fluoroquinolone use. However, the use of cefixime at suboptimal doses at the turn of the century has led to the emergence of cephalosporin resistance, which has spread internationally [6].

All documented gonococcal resistance to cephalosporins to date has been chromosomally-mediated. However, a β-lactamase-producing plasmid has been reported in China, Japan and Thailand [67,68,69], probably acquired from Enterobacteriaceae, which is just a one point mutation away from encoding for an extended-spectrum β-lactamase. If this mutation occurred, it would confer plasmid-mediated resistance, which could spread to different lineages of *N. gonorrhoeae*, potentially leading to the end of cephalosporins as effective anti-gonococcal treatments [6].

GRASP began testing for cefixime resistance in 2004 in advance of the update in treatment guidelines for uncomplicated gonorrhoea, which in 2005 changed from recommending ciprofloxacin to a single dose of either 400 mg cefixime, 250 mg ceftriaxone or 2 g spectinomycin [41,70]. Very little resistance was detected until 2008; however, by 2010, the total level of resistance had passed the 5% threshold at which the WHO recommends that first-line treatment guidelines should be changed [51,65] (Figure 4D). The majority of the resistance was concentrated in the MSM population, where more than one in three isolates exhibited cefixime MIC ≥ 0.125 mg/L in 2010 [48]. This evidence, combined with increasingly-common reports of cefixime treatment failure in the UK, formed the basis of the decision in May 2011 to update treatment guidelines for uncomplicated gonorrhoea [71,72]. Cefixime was no longer recommended as a first-line treatment and was replaced with a combination of 500 mg ceftriaxone and 1 g azithromycin [7]. The proportion of isolates with cefixime MIC ≥ 0.125 mg/L fell concomitantly with prescriptions after its removal as a first-line treatment, suggesting a significant fitness cost to resistance [73].

In spite of this encouraging decline in resistance, an examination of the cefixime MIC values for sensitive isolates reveals that they have been drifting upwards in recent years (Figure 4E). In 2011, one in three isolates had cefixime MIC ≥ 0.015 mg/L, rising to three-quarters by 2015 [52]. The bimodal distribution of the cefixime MIC of isolates from MSM in 2014 suggests that strains with reduced sensitivity continue to circulate among the population (Figure 4F) [51], probably as a result of the widespread recent reliance on other cephalosporins, in particular ceftriaxone.

### 2.5. Ceftriaxone

Ceftriaxone has been one of the recommended treatments for gonorrhoea in the UK since 2005 and in 2011 became the first-line treatment as part of a dual therapy with azithromycin [7,70]. In 2016, it was prescribed in 95% of cases [53]. Ceftriaxone-resistant *N. gonorrhoeae* has been very rarely detected in UK surveillance. A total of 17 cases were found, sporadically, between 2005 and 2013 [46,47,48,49,50]. Ceftriaxone-resistant gonorrhoea was confined to MSM until 2013, with the exception of a single case detected in a heterosexual man in 2009. However, the three ceftriaxone-resistant isolates detected in 2013 were found in heterosexuals.

**Figure 5:**
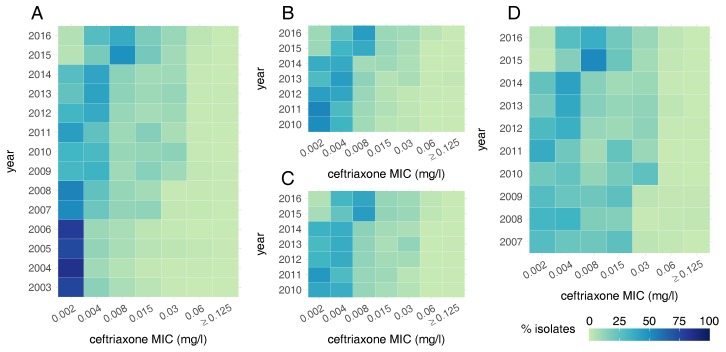
Heat maps showing the ceftriaxone MIC distribution of *N. gonorrhoeae* in the UK over time: (**A**) from all GRASP samples; (**B**) in women; (**C**) in heterosexual men and (**D**) in MSM.

Despite the low levels of ceftriaxone-resistant gonorrhoea in the UK, there has been a considerable reduction in sensitivity to the antibiotic, known as MIC drift [74]. In 2003, more than 75% of isolates tested as part of GRASP were completely sensitive to ceftriaxone (MIC ≤ 0.002 mg/L), but by 2016, the proportion had decreased to less than 5% (Figure 5A) [44,53]. During the cefixime-resistant outbreak in MSM in 2010–2011, the ceftriaxone MIC distribution was distinctly bimodal, due to the outbreak strain having decreased susceptibility to cephalosporins. Isolates from MSM are less sensitive to ceftriaxone than those found in heterosexuals on average, with around one in three isolates from MSM having ceftriaxone MIC ≥ 0.015 mg/L in 2016, compared to around one in five from heterosexuals (Figure 5B–D). However, the modal MIC observed in heterosexuals is increasing, from 0.002 mg/L in 2010 to 0.008 mg/L in 2016 [53].

### 2.6. Azithromycin

Azithromycin was developed in 1980 and found to be efficacious against *N. gonorrhoeae*, as well as other sexually-transmitted bacterial infections, including *Chlamydia trachomatis* [6]. By the latter half of the 1990’s, reports from Latin America, where azithromycin was widely used, suggested that resistance had developed [75]. Indeed, the use of azithromycin to treat chlamydial co-infection may have contributed to the development of gonococcal resistance by exposure to a lower-than-effective dose. One gram of azithromycin is effective in the treatment of chlamydia, whereas 2 g are required to treat gonorrhoea in a single-dose, although the higher dose is commonly associated with a range of gastrointestinal side-effects [76].

**Figure 6:**
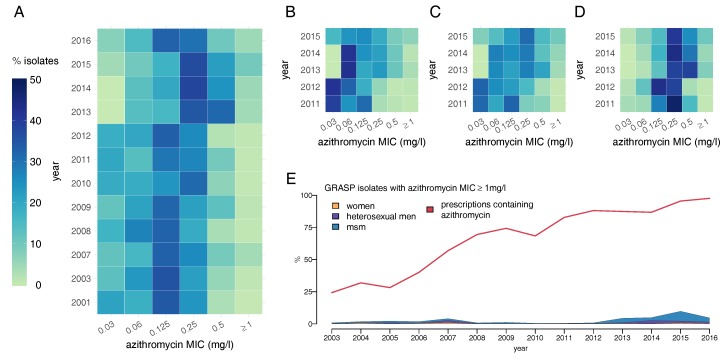
Heat maps showing the azithromycin MIC distribution of *N. gonorrhoeae* in the UK over time: (**A**) from all GRASP samples; (**B**) in women; (**C**) in heterosexual men and (**D**) in MSM; (**E**) Proportion of gonococcal isolates in GRASP showing resistance to azithromycin over time, split by gender and male sexual behaviour compared with the proportion of prescriptions that include azithromycin. Note that the MIC values in 2013 and 2014 have been adjusted upwards by one dilution from those published in GRASP to compensate for poor growth on the agar medium [54].

Azithromycin resistance has become a grave concern; the inclusion of azithromycin in current dual therapy is intended to prevent the emergence of ceftriaxone resistance, which would result in the loss of the last regimen suitable for general first-line use. GRASP began testing for azithromycin resistance in 2001, setting the threshold at MIC ≥ 1 mg/L. Before if became a component of the first-line dual-therapy in 2011, it was commonly used to treat patients who were co-infected with chlamydia or as a single-dose therapy when practitioners judged that a patient was unlikely to return to the clinic for follow-up [44]. Azithromycin resistance has been detected at low levels across all gender and sexuality groups throughout the duration of GRASP (Figure 6E). A surge in resistance was seen in 2007 when 5% of isolates from men, regardless of their sexuality, had resistant strains. There is compelling evidence that many of these infections were imported from the Indian subcontinent [44]. Resistance levels in 2013 and 2014 were underestimated due to problems with the culture medium and were in fact close to 5% [54].

There has been some drift towards resistance in the azithromycin MIC distribution between 2001 and 2016, from one in four isolates having MIC ≥ 0.5 mg/L to one in two (Figure 6A) [53,77]. Between 2011 and 2015, MSM had been infected on average with less susceptible gonococcal strains compared with heterosexuals (Figure 6B–D).

Isolates of highly azithromycin-resistant *N. gonorrhoeae* (MIC ≥ 256 mg/L) were first observed in the UK in 2004 with an outbreak in Scotland, which spread to England and Wales in 2007 [77,78]. This was followed in 2015 by an outbreak of high-level resistance in young heterosexuals in Leeds [11]. The outbreak spread south to London, where the majority of cases were recorded among MSM [79]. Between November 2014 and February 2017, a total of 70 cases were recorded across England [12]. The outbreak strain was characterised by mutations in the macrolide target, 23S rRNA, which was hypothesised to have evolved from low-level resistance due to selection pressure from previous azithromycin exposure [12].

### 2.7. Spectinomycin

Spectinomycin was developed in the early 1960’s as a specific treatment for gonorrhoea and was commonly used for the treatment of penicillin-resistant strains [57]. While it is effective against genital and rectal infections, the efficacy of spectinomycin in treating pharyngeal gonorrhoea has been estimated at only 80% [80].

Resistance first emerged as early as 1967 in the Netherlands [81]. There is evidence that spectinomycin resistance is easily evolved and selected for: its use as a first-line monotherapy among U.S. servicemen in the Republic of Korea in 1981 resulted in the rapid emergence of resistance [82]. Spectinomycin-resistant *N. gonorrhoeae* was first detected in the UK in 1983 [83]. It was still prevalent at the time of the first GRASP report in 2000, which indicated that two isolates were found to have MIC ≥ 128 mg/L [37]. Resistance has since remained rare in the UK; reported cases have been sporadic and mostly from London: one in 2001 and a further three in 2004 [38,41].

### 2.8. Multidrug Resistance

Increasingly, many strains of *N. gonorrhoeae* are becoming resistant to multiple antibiotics simultaneously [2,84]. The first Extensively Drug-Resistant (XDR) strain H041 was isolated in 2009 in Kyoto, Japan, from the throat of a female sex worker [85,86]. This isolate was resistant to the seven clinically-relevant antibiotics mentioned throughout this review, with the exception of spectinomycin. Relatively few XDR *N. gonorrhoeae* have been reported since, suggesting that the high resistance of these strains comes at a high fitness cost, although they might only be a few compensatory mutations away from reducing this cost [36,73], in which case, treating gonorrhoea would become extremely challenging [2,3,6].

The UK GRASP programme reports mostly on resistance to each antibiotic separately, with relatively little information being given on multidrug resistance. Between 2004 and 2015, the proportion of GRASP isolates resistant to more than one clinically-relevant antibiotic increased from 7.5% to 17.5%, with many more strains exhibiting elevated MICs to both azithromycin and ceftriaxone [54]. A retrospective survey of gonorrhoea resistance in Brighton over the same time frame found that 12% of isolates were resistant to at least two of the four antibiotics cefixime, penicillin, azithromycin and ciprofloxacin [87,88].

The first reported case worldwide of dual therapy failure occurred in December 2014 in a British man reporting heterosexual contact in Japan [13]. Test of cure revealed that the treatment had been successful at clearing urogenital infection, but unsuccessful against the pharyngeal infection. Susceptibility testing confirmed that the strain was resistant to ceftriaxone and azithromycin, as well as all other antibiotics reviewed here, except spectinomycin. The individual was eventually successfully retreated by doubling the doses of both ceftriaxone and azithromycin [13]. In February 2018, a similar case was reported of a British man having contracted gonorrhoea in South-East Asia, which testing suggested was susceptible only to spectinomycin. However, test of cure revealed that even this last-line therapy had failed to eradicate the infection in the pharynx [55]. The case was eventually cured after three days of therapy with IV ertapenem [14,89].

## 3. Concluding Remarks

The recent cases of *N. gonorrhoeae* persistent multidrug-resistant pharyngeal infection underline the point that to understand the dynamics of transmission and resistance, we first need to quantify the relative roles of symptomatic versus asymptomatic infection at different anatomical sites. The successful treatment of pharyngeal infections may require higher antibiotic concentrations, and exposure to insufficient doses may be a driver of resistance [90]. Recent work has suggested that pharyngeal infection may be much more important in transmission than previously thought, particularly in MSM [91], although this may also apply to heterosexual transmission [92]. Furthermore, the pharynx may also be an important anatomical site in the emergence and persistence, as well as propagation of resistance [92]. Monitoring all anatomical sites of infection is therefore important.

There are a range of questions about how surveillance of gonorrhoea could be enhanced to improve detection of the emergence and propagation of resistance and to assess the impact of public health interventions when applied at scale in real-world conditions [90,93]. To maximise our ability to interpret sexually-transmitted infection surveillance data to understand transmission patterns, it is important to have information on patients’ recent sexual risk behaviour and reasons for being tested: due to symptoms, routine screening, screening after risky behaviour or being a notified partner [94,95].

With limited resources available for surveillance, they need to be optimally targeted. The UK has a comprehensive surveillance program of sexual health services, with GRASP providing targeted enhanced surveillance of gonorrhoea antibiotic resistance. GRASP provides a rich dataset, but it has limitations, including having sampling for a limited period of each year. There may be a benefit in focusing enhanced surveillance efforts on particular risk groups (for example, MSM or heterosexuals with risky behaviour) and/or geographic locations [90]. Decisions may be assisted by insights into transmission patterns offered by whole genome sequencing [12,33,87,96].

When combined with surveillance and epidemiological data using mathematical modelling techniques [33,90], whole genome sequencing offers important insights into transmission patterns and can inform about the mechanisms of emergence, persistence and propagation of resistance, as well as providing information on the impact of public health interventions. In the medium term, whole genome sequencing is likely to become routine in clinical care, but since it is currently still too expensive (and slow) for such use, further work is required to determine how to optimally select isolates for sequencing. In the short term, point-of-care testing for gonorrhoea is likely to be introduced and to offer identification of known genetic determinants of resistance. This could facilitate the expansion of surveillance for drug resistance, with greater volumes of testing being performed across more geographic locations and on a year-round basis. It will be important to assess how well any point-of-care testing that is introduced works in combating resistance and informing prescribing.

We need a better understanding of the evolutionary fitness benefits and costs of resistance to inform future antibiotic prescribing strategies, including optimal use of new antibiotics [97] and consideration of approaches more sophisticated than just having a universal default first-line therapy [73]. This requires better prescribing data, ideally linked to test results and treatment outcomes at the individual level, so that the selective pressure can be quantified by time, location and patient group. In addition, whole genome sequence data have an important role to play, including in understanding the effects of the genetic background: the same mutation may have a different impact on evolutionary fitness in different lineages. Surveillance has been essential to responding to antimicrobial resistance in gonorrhoea in the UK, but in the context of the escalating epidemic, it is more important than ever to enhance our surveillance efforts and take advantage of new technology to face future challenges.

## Figures and Tables

**Table 1:** EUCASTDefinitions of antimicrobial resistance.

Antimicrobial	Resistance Definition
Ceftriaxone	MIC ≥ 0.25 mg/L
Azithromycin	MIC ≥ 1 mg/L
Cefixime	MIC ≥ 0.25 mg/L
Ciprofloxacin	MIC ≥ 0.125 mg/L
Spectinomycin	MIC ≥ 128 mg/L
Penicillin	MIC ≥ 2 mg/L or β-lactamase positive
Tetracycline	MIC ≥ 2 mg/L
